# Comparison of Current-Use Pesticide and Other Toxicant Urinary Metabolite Levels among Pregnant Women in the CHAMACOS Cohort and NHANES

**DOI:** 10.1289/ehp.0901568

**Published:** 2010-02-03

**Authors:** Rosemary Castorina, Asa Bradman, Laura Fenster, Dana Boyd Barr, Roberto Bravo, Michelle G. Vedar, Martha E. Harnly, Thomas E. McKone, Ellen A. Eisen, Brenda Eskenazi

**Affiliations:** 1 Center for Children’s Environmental Health Research, School of Public Health, University of California, Berkeley, California, USA; 2 Division of Environmental and Occupational Disease Control, California Department of Public Health, Richmond, California, USA; 3 National Center for Environmental Health, Centers for Disease Control and Prevention, Atlanta, Georgia, USA; 4 Lawrence Berkeley National Laboratory and University of California, Berkeley, California, USA

**Keywords:** ETU, exposure, NHANES, organophosphate, pesticides, pregnancy, prenatal, urinary metabolites, women

## Abstract

**Background:**

We measured 34 metabolites of current-use pesticides and other precursor compounds in urine samples collected twice during pregnancy from 538 women living in the Salinas Valley of California, a highly agricultural area (1999–2001). Precursors of these metabolites included fungicides, carbamate, organochlorine, organophosphorus (OP), and pyrethroid insecticides, and triazine and chloroacetanilide herbicides. We also measured ethylenethiourea, a metabolite of the ethylene-bisdithiocarbamate fungicides. Repeat measurements of the compounds presented here have not been reported in pregnant women previously. To understand the impact of the women’s regional environment on these findings, we compared metabolite concentrations from the CHAMACOS (Center for the Health Assessment of Mothers and Children of Salinas) cohort with U.S. national reference data for 342 pregnant women sampled by the National Health and Nutrition Examination Survey (1999–2002).

**Results:**

The eight metabolites detected in > 50% of samples [2,4-dichlorophenol (2,4-DCP); 2,5-dichlorophenol (2,5-DCP); 1- and 2-naphthol; *ortho*-phenylphenol (ORTH); *para*-nitrophenol (PNP); 2,4,6-trichlorophenol (2,4,6-TCP); and 3,4,6-trichloro-2-pyridinol (TCPy)] may be related to home or agricultural pesticide use in the Salinas Valley, household products, and other sources of chlorinated phenols. More than 78% of women in this study had detectable levels of at least one of the OP pesticide-specific metabolites that we measured, and > 30% had two or more. The 95th percentile values of six of the most commonly detected (> 50%) compounds were significantly higher among the CHAMACOS women after controlling for age, race, socioeconomic status, and smoking [(2,4-DCP; 2,5-DCP; ORTH; PNP; 2,4,6-TCP; and TCPy); quantile regression *p* < 0.05].

**Conclusions:**

Findings suggest that the CHAMACOS cohort has an additional burden of precursor pesticide exposure compared with the national sample, possibly from living and/or working in an agricultural area.

Prenatal exposure to pesticides, including organophosphorus (OP) and organochlorine (OC) compounds, has been shown to increase the risk of adverse developmental outcomes in children (Eskenazi et al. 2008; Jurewicz and Hanke 2008; Perera et al. 2005). However, few studies have comprehensively investigated maternal exposures to currently used pesticides during pregnancy. Biomonitoring results from the National Health and Nutrition Examination Survey (NHANES) (1999–2002) [Centers for Disease Control and Prevention (CDC) 2005] and other studies suggest that many pregnant women are exposed to a mixture of insecticidal and fungicidal compounds (Berkowitz et al. 2003; Bradman et al. 2003, 2005; Takser et al. 2004; Ye et al. 2008).

There are multiple pesticide exposure pathways, but recent research (Lu et al. 2006; McKone et al. 2007) suggests that diet is the dominant route of exposure for the general population. Para-occupational or take-home exposure from farmworkers and ambient exposure from living in proximity to agricultural activity can add to total pesticide exposure in agricultural communities (Goldman et al. 2004; McKone et al. 2007). Biomonitoring can help to quantify the additional exposure for pregnant women in agricultural communities by allowing for the direct comparison of detection frequencies and metabolite levels between populations.

The Center for the Health Assessment of Mothers and Children of Salinas (CHAMACOS) is a longitudinal birth cohort study investigating *in utero* and postnatal environmental exposures and their effects on the health of children residing in the largely agricultural Salinas Valley of Monterey County, California (Eskenazi et al. 2003). The growing season in this productive region is nearly year-round. Row crops are grown with a predominance of strawberries, lettuce, and broccoli in the north and warmer-weather crops such as grapes in the south. Previously we presented CHAMACOS prenatal measurements of three specific metabolites of OP pesticides (Eskenazi et al. 2004) and ethylenethiourea (ETU), a toxic metabolite of the ethylene bisdithiocarbamate (EBDC) fungicides (Montesano et al. 2007). In this study, we present exposure data on an additional 30 pesticide metabolites in repeat samples collected from pregnant women late in the first and second trimesters. To better understand the impact of the regional agricultural environment on these findings, we also compare levels in the CHAMACOS cohort with those measured in a representative national sample of pregnant women participating in the NHANES (1999–2002) (CDC 2005).

## Methods

### Study location characteristics

The Salinas Valley includes approximately 91,054 irrigated hectares devoted to row, vineyard, and orchard crops. In 2000, approximately 570,000 kg herbicide, EBDC fungicide, and carbamate, OP, OC, and pyrethroid pesticide active ingredients were applied for agricultural and landscaping purposes in this region, a level typical of recent years (10% herbicides; 27% EBDC fungicides; 44% OP and 2% OC pesticides; and 4% pyrethroid pesticides) [California Department of Pesticide Regulation (CDPR) 2000a, 2006]. Of the total kilograms of nonfumigant pesticides applied in the Salinas Valley (excluding sulfur and petroleum products), 65% percent devolve into one of the 34 urinary metabolites we measured. Applications of maneb, diazinon, malathion, acephate, and chlorpyrifos account for 78% of the kilograms of pesticides applied whose urinary metabolites were detectable using the analytic methods available in this study (Table 1).

### Study population

#### CHAMACOS

Between September 1999 and November 2000, 601 pregnant women were enrolled in the CHAMACOS birth cohort study from six local prenatal clinics. Women were eligible to participate in this study if they were ≤ 20 weeks gestation at the time of enrollment, were ≥ 18 years of age, qualified to receive poverty-based government health insurance, and planned to continue receiving prenatal care at a participating health center (Eskenazi et al. 2003, 2004). Written informed consent was obtained from all participants in accordance with procedures approved by the University of California Berkeley Committee for the Protection of Human Subjects.

#### NHANES 1999–2002 data set

As part of the ongoing NHANES, the CDC reported levels of current-use pesticide metabolites measured in spot urine collected from representative samples of the U.S. population stratified by age, sex, and racial/ethnic group (Barr et al. 2005; CDC 2004). Metabolite concentrations were measured in 3,048 urine samples collected from U.S. residents 6–59 years of age during 1999 and 2002 and included 342 pregnant women 15–50 years of age (CDC 2004). The public release versions of the NHANES data sets, including demographic information and metabolite data, were used for these analyses (CDC 2007a, 2007b). We applied no sample weights to the NHANES data.

### Pesticide use reporting data

In California, all agricultural, right-of-way, and structural pesticides applied by farmers or licensed applicators must be reported to the state (CDPR 2000b). Agricultural pesticide applications, representing 90% of all reported use, are geocoded to 1-square-mile units based on the Public Land Survey System (PLSS). To summarize agricultural pesticide use in the Salinas Valley, we first defined the region as the area bounded by the Pacific Ocean and an isopleth at 200 feet in elevation above the Salinas River. We then identified the PLSS sections within the Salinas Valley and abstracted pesticide use from the California pesticide use reporting (PUR) data set.

### Study interview

Information on demographics, health, household characteristics, and occupation of CHAMACOS participants was collected through personal interviews. Interviews were conducted in English or Spanish by bilingual, bicultural study staff. The first prenatal interview occurred shortly after enrollment in the study (mean ± SD = 13 ± 5.2 weeks gestation) and a follow-up interview occurred late in the second trimester (mean = 26 ± 2.6 weeks gestation).

### Urine collection and analysis

Two spot urine samples were collected from CHAMACOS participants at each prenatal interview and analyzed by the Division of Laboratory Sciences, CDC. Women voided into a sterile urine cup in bathroom facilities at our field office or in a mobile clinic. Specimens were placed into precleaned glass containers with Teflon-lined caps, bar coded, and stored at −80°C until shipment. Samples were shipped on dry ice to the CDC and stored at −70°C until analysis.

### Pesticide and creatinine measurement

Thirty-four urinary metabolites were measured for the CHAMACOS cohort, including OP, OC, and pyrethroid pesticides, herbicides, and EBDC fungicides. Acephate (AP), methamidophos (MMP), omethoate (OMT), dimethoate (DME), and the metabolites ETU and propylenethiourea (PTU) are water soluble and after lyophilization were extracted with dichloromethane; they were then analyzed using isotope dilution high-performance liquid chromatography with atmospheric pressure chemical ionization tandem mass spectrometry (HPLC/APCI-MS/MS) (Montesano et al. 2007). Sample preparation for the phenolic metabolites [2,4-dichlorophenol (2,4-DCP); 2,5-dichlorophenol (2,5-DCP); carbofuranphenol (CFP); 1-naphthol; 2-naphthol; *para*-nitrophenol (PNP); pentachlorophenol (PCP); *ortho*-phenylphenol (ORTH); 2,4,5-trichlorophenol (2,4,5-TCP); 2,4,6-trichlorophenol (2,4,6-TCP); and 3,5,6-trichloro-2-pyridinol (TCPy)] involved enzyme hydrolysis to liberate them from their glucuronide or sulfate conjugates, isolation of the target chemicals using solid phase extraction cartridges, a phase-transfer catalyzed derivatization, cleanup using sorbent-immobilized liquid/liquid extraction cartridges, and concentration of the sample. Derivatized samples were analyzed by capillary gas chromatography- tandem mass spectrometry using isotope dilution calibration for quantification (Bravo et al. 2005). The remaining specific metabolites of OP pesticides [5-chloro-1,2-dihydro-1- isopropyl-[3H]-1,2,4-triazol-3-one (CIT); 3-chloro-4-methyl-7-hydroxycoumarin (CMHC); 2-diethylamino-6-methylpyrimidin-4-ol (DEAMPY); 2-isopropyl-4-methyl-6-hydroxypyrimidinol (IMPY); malathion dicarboxylic acid (MDA)]; synthetic pyrethroids; and diethyl-m-toluamide (DEET) were measured as the intact metabolite after enzyme hydrolysis and solid phase extraction using HPLC/APCI-MS/MS (modification of Olsson et al. 2004). Creatinine concentrations in urine were determined using a commercially available diagnostic enzyme method (Vitros CREA slides; Ortho Clinical Diagnostics, Raritan, NJ). The same analytical laboratory at CDC performed the urinary metabolite measurements for both the NHANES and CHAMACOS studies (CDC 2004) (Table 1).

Laboratory quality control (QC) included repeat analysis of three in-house urine pools enriched with known amounts of pesticide residues whose target values and confidence limits were previously determined (Westgard 2003). Detection limits ranged from 0.03 to 1.5 μg/L. We assigned an imputed value of the limit of detection (LOD)/✓2– to levels below the detection limit (Barr et al. 1999; Hornung and Reed 1990). A total of 153 (blind) field QC samples were analyzed, representing 15% of total samples. Mean relative recoveries for the metabolites in field QC samples ranged from 78 to 120%. The mean levels of metabolites in 51 blank field samples were ≤ 1 μg/L.

The creatinine concentration in each urine sample was reported in milligrams of creatinine per deciliter of urine. Of the 601 women who enrolled in the study, adequate urine samples with valid creatinine levels were collected from 538 (90%) women at the first prenatal sampling point and 481 (80%) women at the second.

### Ratio of 1-naphthol to 2-naphthol

Both naphthalene and carbaryl can devolve to urinary 1-naphthol. Because naphthalene, but not carbaryl, also metabolizes to 2-naphthol, the ratio of urinary 1-naphthol to 2-naphthol within an individual can provide information about the source of 1-naphthol. Thus, we calculated the ratio of 1-naphthol to 2-naphthol to identify subgroups within the CHAMACOS cohort and pregnant women in NHANES in which carbaryl was expected to be the primary source of 1-naphthol. We assumed that a ratio > 2 indicates carbaryl as the source of 1-naphthol (Meeker et al. 2007).

### Data analysis

We calculated descriptive statistics for metabolite levels from the two CHAMACOS prenatal urine samples. For the eight CHAMACOS metabolites with detection frequencies > 50% in both samples, we computed the Spearman’s rank correlation between the first and second prenatal samples. In addition, we computed metabolite correlations within each of the two CHAMACOS samples and within samples collected from pregnant women in NHANES. To broadly compare the NHANES and CHAMACOS metabolite levels, we analyzed the 11 compounds with detection frequencies > 40% in either study. We used the Wilcoxon rank-sum test and quantile regression (95th percentile) to compare differences in metabolite concentrations between the CHAMACOS cohort and NHANES. We compared differences in detection frequencies using analysis of variance (ANOVA). For compounds with LODs that differed between the two populations (e.g., 1- and 2-naphthol), the higher LODs were applied to the metabolite concentrations of both populations for analyses of differences in detection frequencies. When comparing NHANES and CHAMACOS women’s metabolite levels using quantile regression, we used demographic variables to adjust our models for age (continuous), current smoking (yes/no), ethnicity (Mexican or Mexican American; other Hispanic; non-Hispanic white; non-Hispanic black; or other race, including multiracial), and socioeconomic status (at or below poverty threshold; within 200% of poverty threshold; above 200% of poverty threshold) (Table 2**)**. Finally, we calculated the total number of OP pesticide metabolites detected in the first and second prenatal urine samples of each CHAMACOS woman. We did not count PNP, because compounds other than OP pesticides (e.g., nitrobenzene) are additional sources of urinary PNP.

All analyses were conducted using Stata software, version 10 (StataCorp LP, College Station, TX).

## Results

### Demographic characteristics

Table 2 presents the demographic characteristics of pregnant women in the CHAMACOS cohort and NHANES. Compared with pregnant women in NHANES, the CHAMACOS women were less educated (80% vs. 23% attained less than a 12th grade education), lived with more people in their households (74% vs. 29% live with ≥ 4 household members), and were poorer (62% vs. 25% living at or below the federal poverty threshold). Ninety-five percent of CHAMACOS women were Mexican or Mexican American compared with 26% in NHANES. Forty-four percent of CHAMACOS women were employed as farm workers at some point during their pregnancy, and 81% percent shared a home with at least one agricultural worker (Eskenazi et al. 2004). In addition, the CHAMACOS women tended to have higher prepregnancy body mass indexes (BMIs) compared with NHANES (23% vs. 15% with BMI > 30, respectively), but fewer CHAMACOS women smoked (1.5%) compared with pregnant women in NHANES (7.4%).

### Creatinine

Median CHAMACOS creatinine levels decreased slightly from the first to the second prenatal sample, with levels of 93.7 mg/dL [interquartile range (IQR) = 51.5–139] and 90.8 mg/dL (IQR = 60.8–129), respectively. Median creatinine levels among pregnant women in CHAMACOS [combined first and second prenatal samples = 92.7 mg/dL (55.8–135)] were significantly lower than those of NHANES [108 mg/dL (IQR = 64.0–153); *p* < 0.05]. In both populations, women’s urinary creatinine levels decreased significantly with increasing weeks of gestation (*p* < 0.05). This trend is consistent with medical reference data (Becker et al. 1992; Davison and Noble 1981; Davison et al. 1980). The NHANES prenatal samples were collected at approximately 24 weeks gestation (mean = 24.4 ± 9.5 weeks).

### Urinary metabolite concentration data

Table 2 describes the 34 analytes measured and the amount of precursor compound active ingredients applied in the Salinas Valley in 2000 (CDPR 2000a). Table 3 presents CHAMACOS and NHANES descriptive statistics for the most frequently detected metabolites (detection frequency > 50%) in the CHAMACOS cohort. The CHAMACOS detection frequencies ranged from 0 to 81%, with eight metabolites detected in both urine samples at frequencies over 50% (2,4- and 2,5-DCP; 1- and 2-naphthol; PNP; ORTH; 2,4,6-TCP; and TCPy). The NHANES detection frequencies for the metabolites ranged from 0 to 100%, with seven metabolites detected at frequencies over 50% [2,4- and 2,5-DCP; 1- and 2-naphthol; 3-phenoxybenzoic acid (3PBA2); 2,4,6-TCP; and TCPy].

Table 4 summarizes the CHAMACOS and NHANES levels of the metabolites with average CHAMACOS detection frequencies < 50%. The CHAMACOS LODs were the same for the first and second prenatal sampling points and, except for 1- and 2-naphthol, were similar to those reported in NHANES (Tables 3 and 4) (CDC 2005). Among compounds with > 40% detection, the CHAMACOS and NHANES detection frequencies were statistically different for 2,5-DCP, 1- and 2-naphthol, ORTH, 3PBA2 and 2,4,5-TCP. The CHAMACOS first and second prenatal 2,5-DCP, ORTH, and 2,4,5-TCP detection frequencies were significantly higher than NHANES, and the NHANES 1- and 2-naphthol and 3PBA2 detection frequencies were significantly higher than CHAMACOS (ANOVA *p* < 0.05).

Spearman correlations between the two CHAMACOS sampling points for the eight most frequently detected metabolites ranged from 0.01 to 0.31. Most correlations were weak (rho ~ 0.15). Moderate correlations were observed for 2,5-DCP (rho = 0.31) and 2-naphthol (rho = 0.26). The intraclass correlation coefficients between the two sampling points for these metabolites ranged from 0.02 to 0.30. Overall, these analyses indicate weak to moderate correlations between the two sampling points for all frequently detected compounds except 1-naphthol and ORTH [see Supplemental Material, Table 1 (doi:10.1289/ehp.0901568)].

The correlations of the eight most frequently detected metabolites within each CHAMACOS sampling time cross-section ranged from weakly negative to strongly positive (Spearman rho = –0.13 to 0.66), indicating that some participants were exposed simultaneously to several pesticides [see Supplemental Material, Table 2 (doi:10.1289/ehp.0901568)]. The strongest correlations were found between 2,4,6-TCP and TCPy within the first and second prenatal samples (rho = 0.66 and rho = 0.42, respectively; *p* < 0.01) and between 2,4-DCP and 2,5-DCP within the first and second prenatal samples (rho = 0.61 and rho = 0.65, respectively; *p* < 0.01). 1-Naphthol and 2-naphthol, which can devolve from naphthalene, were moderately correlated (rho = 0.40 and 0.55 within the first and second prenatal samples, respectively; *p* < 0.01). Similarly, in NHANES, 2,4,6-TCP and TCPy (rho = 0.55) and 2,4-DCP and 2,5-DCP (rho = 0.53) were strongly correlated (*p* < 0.01). 1-Naphthol and 2-naphthol levels were also significantly correlated (rho = 0.56; *p* < 0.01) in NHANES. Overall, the patterns of within-sample correlations were similar between CHAMACOS and NHANES. In all cases except PNP, when two metabolites were correlated in NHANES, the same metabolites were also significantly correlated in one or both of the CHAMACOS prenatal samples.

In the CHAMACOS cohort, the detection frequency of ETU was higher at the first (23.5%) compared with the second (7.7%) sampling time. In addition, the 95th percentile value of ETU was significantly higher in the first compared with the second prenatal sample (1.5 μg/L vs. 0.4 μg/L, respectively; *p* < 0.01).

### Comparison of CHAMACOS and NHANES metabolite levels

Results of the Wilcoxon rank-sum test suggest that for six (2,5-DCP; MDA; 2-naphthol; 3PBA2; 2,4,5-TCP; and 2,4,6-TCP) of 11 frequently detected metabolites (i.e., detection frequencies > 40%), there were statistically significant differences between the distributions of the CHAMACOS first prenatal samples and NHANES metabolite levels (*p* < 0.05). Among these six compounds, the distributions of four (2,5-DCP; MDA; 2,4,5-TCP; and 2,4,6-TCP) of the first prenatal CHAMACOS metabolite levels were higher compared with those in NHANES, and the distributions of two NHANES metabolite levels (2-naphthol and 3PBA2) were higher compared with those in CHAMACOS.

In addition, there were statistically significant differences between the distributions of eight (2,5-DCP; MDA; 2-naphthol; PNP; 3PBA2; 2,4,5-TCP; 2,4,6-TCP; and TCPy) of the 11 frequently detected metabolites in the CHAMACOS second sample versus the NHANES metabolite levels (*p* < 0.05). Among these eight compounds, the distributions of six (2,5-DCP; MDA; PNP; 2,4,5-TCP; 2,4,6-TCP; and TCPy) of the second prenatal CHAMACOS metabolites were higher compared with those in NHANES, and the distributions of two NHANES metabolite levels (2-naphthol and 3PBA2) were higher compared with those in CHAMACOS. The NHANES prenatal samples were collected at approximately 24 weeks gestation, and the CHAMACOS first and second prenatal samples were collected at approximately 13 and 26 weeks gestation, respectively. Thus, the CHAMACOS second prenatal metabolite levels may be more comparable with those from pregnant women in NHANES.

We compared median concentrations of the six compounds with detection frequencies > 50% in both populations using quantile regression and adjusting for age, race, socioeconomic status, and smoking. The CHAMACOS first prenatal median 2,4-DCP and 2,5-DCP concentrations were higher than NHANES (*p* < 0.05). Further, the CHAMACOS second prenatal median 2,5-DCP, 2,4,6-TCP, and TCPy concentrations were also higher than NHANES (*p* < 0.05). By contrast, the NHANES 2-naphthol median levels were significantly higher than the CHAMACOS first and second prenatal levels (*p* < 0.05) (Table 3).

**Figure 1:**
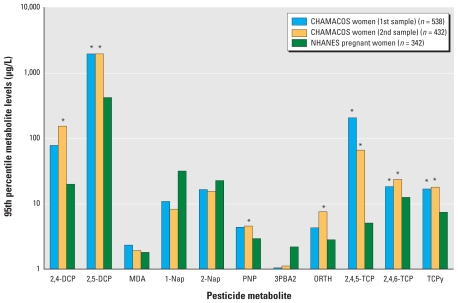
CHAMACOS first and second prenatal 95th percentile metabolite levels compared with pregnant women in NHANES 1999–2002. Nap, naphthol. *95th percentile quantile regression adjusting for age, race, socioeconomic status, and smoking; *p* < 0.05.

Figure 1 presents a comparison of the 95th percentile metabolite values for the CHAMACOS cohort (both first and second prenatal samples) and NHANES for the 11 metabolites detected at > 40% in either study. Using quantile regression, the CHAMACOS first and/or second prenatal 95th percentile values were statistically higher compared with NHANES for seven of the 11 metabolites. These seven included six of the most commonly detected metabolites (2,4-DCP; 2,5-DCP; ORTH; PNP; 2,4,6-TCP; and TCPy). [Probability plots examining the *z*-scores of CHAMACOS vs. NHANES TCPy, 2,4,5-TCP, and 2,5-DCP levels are presented in the Supplemental Material (doi:10.1289/ehp.0901568).] We found no significant differences between the CHAMACOS first and second prenatal 95th percentile metabolite levels presented in Figure 1.

A metabolite of several commonly used pyrethroids (3PBA2), including permethrin, cypermethrin, cyhalothrin, deltamethrin, and fenvalerate, was detected in urine samples of 67% of NHANES pregnant women compared with 21% and 27% in the CHAMACOS first and second prenatal samples, respectively. In addition, the CHAMACOS 3PBA2 95th-percentile metabolite levels were lower than the NHANES level, albeit not significantly (Figure 1). We could not compare the CHAMACOS and NHANES metabolite levels of several high-use precursor pesticides (dimethoate, acephate, methamidophos, and maneb) because they have not yet been reported in NHANES.

**Figure 2:**
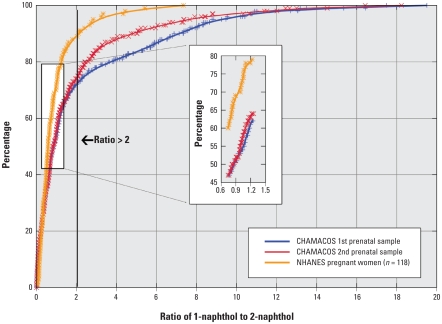
Cumulative distributions of urinary 1-naphthol to 2-naphthol ratios for the CHAMACOS cohort and pregnant women in NHANES 2000–2001.

Figure 2 presents the cumulative distributions of urinary 1-naphthol to 2-naphthol ratios in the CHAMACOS cohort and NHANES. Ratios > 2 occurred in 22% and 24% of CHAMACOS first and second prenatal samples compared with 13% among pregnant women in NHANES. Conversely, the frequency of 1-naphthol to 2-naphthol ratios in the region near unity (i.e., ratios ranging from 0.75 to 1.25) were higher among women in NHANES (32%) compared with the CHAMACOS women at the first and second prenatal sampling times (19% and 20%, respectively) (Figure 2 inset).

## Discussion

We measured 34 specific metabolites in two urine samples collected at approximately 13 and 26 weeks gestation from 538 pregnant women living in an agricultural area, including tests for fungicides, carbamate, OC, OP and pyrethroid insecticides, triazine and chloroacetanilide herbicides, and several chlorinated phenol compounds. To better understand the impact of the regional environment on these metabolite levels in the CHAMACOS cohort, we compared urinary metabolite concentrations from the CHAMACOS cohort with U.S. national reference data for 342 pregnant women sampled by NHANES (1999–2002).

Overall, our results suggest that this population is chronically exposed to several current-use pesticides and intermittently exposed to others. Between 78% and 86% of CHAMACOS women had urine samples with biomarkers for at least one OP pesticide, and 30–34% were exposed to two or more compounds. These findings are consistent with earlier data that we reported indicating that the nonspecific OP dialkylphosphate metabolites were present in > 90% of CHAMACOS women, with levels significantly higher than those found for women in NHANES 1999–2000 (Bradman et al. 2005).

Other studies comparing biomonitoring data with NHANES have found that population-specific factors may explain higher metabolite levels or detection frequencies (Bradman et al. 2005, 2007; Fenske et al. 2005). Of 26 possible comparisons between CHAMACOS and NHANES urinary metabolite levels, detection frequencies were > 50% for just seven metabolites measured in NHANES and eight measured in CHAMACOS. Notably, six of the frequently detected compounds were common to both studies (2,4-DCP; 2,5-DCP; 1-naphthol; 2-naphthol; 2,4,6-TCP; and TCPy). These metabolites devolve from chlorpyrifos; carbaryl; naphthalene; lindane [γ-hexachlorocyclohexane (γ-HCH)]; 1,3- and 1,4-dichlorobenzene; and PCP (Table 1), all relatively persistent in the environment. The increase in CHAMACOS concentrations at the 95th percentile for the chlorinated phenol compounds (2,4- and 2,5-DCP and 2,4,6-TCP) is likely contributed to by population-specific but nonagricultural factors such as proximity to manufacturing or waste incineration, and household product choices (e.g., mothballs, cleaning detergents, and head lice treatments). The similarity of OP pesticide metabolite detection frequencies between the CHAMACOS cohort and a U.S. national reference population support previous multimedia, multipathway modeling efforts that suggest that diet is a major route of exposure in both populations (McKone et al. 2007).

In our analysis, we have limited our metabolite level comparisons with NHANES to pregnant women. By doing so, we compare the CHAMACOS cohort with a national sample of woman also experiencing the changes in diet and metabolism that accompany pregnancy. Furthermore, through this research we have expanded the number of pesticide-specific metabolites reported in pregnant women and provided repeat measurements from samples collected at consistent prenatal time points. Many of the compounds presented here have not been measured or reported before in pregnant women.

Human exposure to naphthalene, a polycyclic aromatic hydrocarbon and component of vehicle exhaust, smoke, and mothballs, is widespread. Carbaryl (n-methylcarbamate insecticide) is commonly used in agriculture. Naphthalene devolves to 1- and 2-hydroxynaphthalene (1- and 2-naphthol) in approximately equal proportions, whereas carbaryl devolves to 1-naphthol only. Thus, a higher ratio of 1-naphthol to 2-naphthol suggests exposure to carbaryl. Median 1-naphthol concentrations in the CHAMACOS cohort were similar to median concentrations among pregnant women in NHANES 2001–2002; however, the median and underlying distribution of the 2-naphthol metabolite were statistically greater in NHANES compared with the CHAMACOS first and second prenatal samples. The higher levels in NHANES may be due to higher smoking rates in pregnant women participating in NHANES (7.4%) compared with CHAMACOS (1.5%). Our finding that 23% of CHAMACOS women had 1-naphthol to 2-naphthol ratios > 2 compared with 13% among pregnant women in NHANES suggests additional sources of carbaryl exposure in the CHAMACOS cohort (Meeker et al. 2007).

ETU is the principal metabolite of the EBDC fungicides (maneb and mancozeb). ETU is more toxic than its precursor compounds (Houeto et al. 1995). More than 150,000 kg of maneb and mancozeb are used annually in the Salinas Valley region, primarily on lettuce and wine grapes (CDPR 2000a). CHAMACOS is the first study to report repeated urinary ETU levels in a U.S. population and in pregnant women. We found that the ETU levels decreased with pregnancy, with higher detection frequencies and levels in samples collected during the first trimester compared with samples collected during the second trimester. We do not know if this change is due to differences in exposure or in metabolism over the course of pregnancy. More information on the metabolism and excretion of these compounds during pregnancy is needed. Urinary ETU levels in the CHAMACOS cohort were similar to those of nonoccupational populations studied in Italy (Aprea et al. 1996; Colosio et al. 2006; Saieva et al. 2004), but lower than those reported for pesticide applicators in other countries (Colosio et al. 2002; Panganiban et al. 2004; Steenland et al. 1997).

Our findings indicate that pregnant women in the Salinas Valley are chronically exposed to several current-use OP and OC pesticides and chlorinated phenols, with additional intermittent exposures to other pesticides. Overall, pesticide metabolite detection frequencies in our population were similar to those found in a U.S. reference population of pregnant women; however, significantly higher 95th percentiles for several metabolites (e.g., PNP; 2,4,5-TCP; TCPy) in our population suggest that a subset of CHAMACOS women experience additional exposures potentially related to regional agricultural pesticide use. The cumulative effect of these exposure levels on pregnant women or their offspring is not known.

## Figures and Tables

**Table 1:** Description of 34 analytes in urine and amount of precursor compound applied in the Salinas Valley in 2000.

Analyte	Possible precursor compound(s) (kilograms active ingredient applied in 2000)^a^	Chemical class	Available in NHANES
Acephate (AP)	AP (33,602)	OP insecticide	No
Acetochlor mercapturate (ACET)	Acetochlor (0)	Herbicide (chloroacetanilide)	Yes
Atrazine mercapturate (ATZ)	Atrazine (4)	Herbicide (triazine)	Yes
Carbofuranphenol (2,3-dihydro-2,2-dimethy-7-hydroxybenzofuran) (CFP)	Carbofuran (7,288); benfuracarb (0); carbosulfan (0)	Carbamate pesticide (n-methyl)	Yes
5-Chloro-1,2-dihydro-1-isopropyl-[3H]-1,2,4-triazol-3-one (CIT)	Isazaphos (0); isazaphos methyl (0)	OP insecticide	No
3-Chloro-4-methyl-7-hydroxycoumarin (CMHC)	Coumaphos (0)	OP insecticide	Yes
*cis*-2,2-(Dibromo)-2-dimethylvinyl cyclopropane carboxylic acid (DBCA)	Deltamethrin (0)	Pyrethroid pesticide	No
*cis*-2,2-(Dichloro)-2-dimethylvinyl cyclopropane carboxylic acid) (*cis*-DCCA)	Permethrin (11,439); cypermethrin (953); cyfluthrin (6)	Pyrethroid pesticide	Yes
*trans*-2,2-(Dichloro)-2-dimethylvinyl cyclopropane carboxylic acid) (*trans*-DCCA)	Permethrin (11,439); cypermethrin (953); cyfluthrin (6)	Pyrethroid pesticide	Yes
2,4-Dichlorophenol (2,4-DCP)	Bifenox (0); chlomethoxyfen (0); 2,4-dichlorophenoxyacetic acid (2,4-D) (0); 2,4-DB (0); 1,3-dichlorobenzene (0); dichlofenthion (0); dichlorprop (0); diclofop (0); nitrofen (0)	Herbicide (chlorinated phenoxy compound)	Yes
2,5-Dichlorophenol (2,5-DCP)	Lindane (γ-HCH) (355); 1,4-dichlorobenzene (0)	Other pesticide (OC)	Yes
2,4-Dichlorophenoxyacetic acid (2,4-D)	2,4-D (and its esters) (0)	Herbicide (chlorinated phenoxy compound)	Yes
2-Diethylamino-6-methylpyrimidin-4-ol (DEAMPY)	Pirimiphos methyl (0)	OP insecticide	Yes
Diethyl-m-toluamide (DEET)	DEET (0)	Other pesticide (repellent)	Yes
Dimethoate (DME)	DME (16,152)	OP insecticide	No
Ethylenethiourea (ETU)	Mancozeb (6,259); maneb (146,592); metiram (0); zineb (0)	EBDC fungicide	No
4-Fluoro-3-phenoxybenzoic acid (4F3PBA)	Cyfluthrin (6)	Pyrethroid pesticide	Yes
2-Isopropoxyphenol (IPP)	Propoxur (0)	Carbamate insecticide	Yes
2-Isopropyl-4-methyl-6-hydroxypyrimidinol (IMPY)	Diazinon (56,051)	OP insecticide	Yes
Malathion dicarboxylic acid (MDA)	Malathion (45,710)	OP insecticide	Yes
Methamidaphos (MMP)	MMP (620); AP (33,602)	OP insecticide	No
o-Methoate (OMT)	OMT (0)	OP insecticide	No
Metolachlor mercapturate (MET)	Metolachlor (1,070)	Herbicide (chloroacetanilide)	Yes
1-Naphthol	Carbaryl (9,190); naphthalene (0)	Carbamate insecticide; PAH	Yes
2-Naphthol	Naphthalene (0)	PAH	Yes
*para*-Nitrophenol (PNP)	Parathion (3); methyl parathion (0); EPN (0); nitrobenzene (0)	OP insecticide; organic compound	Yes
Pentachlorophenol (PCP)	γ-HCH (355); PCP (0)	OC pesticide (fungicide/ wood preservative)	Yes
3-Phenoxybenzoic acid (3PBA2)	General pyrethroid metabolite (14,938)	Pyrethroid pesticide	Yes
*ortho*-Phenylphenol (ORTH)	ORTH (0)	Other pesticide (fungicide)	Yes
Propylenethiourea (PTU)	Propineb (0)	EBDC fungicide	No
2,4,5-Trichlorophenol (2,4,5-TCP)	γ-HCH (355); chlorinated benzenes (—); chlorinated phenols (—)	OC pesticide	Yes
2,4,6-Trichlorophenol (2,4,6-TCP)	γ-HCH (355); chlorinated benzenes (—); chlorinated phenols (—)	OC pesticide	Yes
2,4,5-Trichlorophenoxyacetic acid (2,4,5-T)	2,4,5-T (0)	Herbicide (chlorinated phenoxy acid)	Yes
3,5,6-Trichloro-2-pyridinol (TCPy)	Chlorpyrifos (26,691); chlorpyrifos methyl (0)	OP insecticide	Yes

Abbreviations: —, not available in the PUR data set. EPN, ethyl p-nitrophenol thiobenzenephosphonate; PAH, polycyclic aromatic hydrocarbon.

a Kilograms active ingredient applied includes agricultural, landscape maintenance, structural pest control, and roadside pesticide usage (CDPR 2000a).

**Table 2:** Demographic characteristics of CHAMACOS cohort and pregnant women participating in NHANES 1999–2002.^a^,^b^

	*n* (%)	
Characteristic	CHAMACOS (*n* = 538)	NHANES^c^ (*n* = 342)
Age (mean ± SD)	26.1 ± 5.2	27.0 ± 5.9
Education		
< 12th grade	429 (79.7)	66 (23.2)
Completed high school/GED	60 (11.2)	66 (23.2)
Some college/technical school	45 (8.4)	79 (27.8)
College grad	4 (0.7)	73 (25.7)
Marital status		
Married/living as married	436 (81.0)	233 (74.7)
Single	102 (19.0)	79 (25.3)
Total no. of people in household		
1–2	21 (4.4)	75 (23.2)
3–4	102 (21.6)	154 (47.7)
> 4	350 (74.0)	94 (29.1)
Parity (no. of live births)		
0	193 (35.9)	20 (6.2)
≥ 1	345 (64.1)	303 (93.8)
Country of birth		
Mexico	458 (85.1)	52 (16.1)
United States	68 (12.6)	239 (74.0)
Other	12 (2.2)	32 (9.9)
Ethnicity		
Mexican or Mexican American	512 (95.2)	84 (26.0)
Other Hispanic	9 (1.7)	20 (6.2)
Non-Hispanic white	8 (1.5)	148 (45.8)
Non-Hispanic black	0 (0)	50 (15.5)
Other race including multiracial	9 (1.7)	21 (6.5)
Poverty		
≤ Poverty threshold	332 (61.8)	75 (25.5)
200% poverty threshold	184 (34.3)	64 (21.8)
> 200% poverty threshold	21 (3.9)	155 (52.7)
Prepregnancy BMI^d^		
Underweight (< 18.5)	3 (0.6)	26 (6.2)
Normal (18.5–24.9)	196 (37.5)	231 (55.1)
Overweight (25–29.9)	205 (39.2)	98 (23.4)
Obese (≥ 30)	119 (22.7)	64 (15.3)
Woman currently smokes		
Yes	8 (1.5)	21 (7.4)
No	529 (98.5)	263 (92.6)
Other household members who smoke		
Yes	38 (7.1)	55 (17.1)
No	496 (92.9)	265 (82.6)
Don’t know	0 (0)	1 (0.3)

a At time of the first CHAMACOS prenatal visit (~ 13 weeks gestation).

b Total number of observations varies because of missing data.

c NHANES mean gestational age (5.8 ± 2.0 months).

d Prepregnancy BMI (kg/m^2^) calculated from women’s self-reported prepregnancy height and weight.

**Table 3:** Summary of eight CHAMACOS first and second prenatal and NHANES urinary metabolite concentrations (μg/L) with detection frequencies > 50%.

Analyte	Prenatal sample	*n*	LOD (μg/L)	DF (%)	Percentile					
					25th	50th	75th	90th	95th	Max
2,4-DCP	1	523	0.2	63.7	< LOD	1.8	12.3	35.5	76.9	5313.6
	2	478		57.5	< LOD	1.1	8.9	37.7	152.6	5948.6
	NHANES	223	0.3	53.4	< LOD	0.29	2.6	7.8	19.8	141.8

2,5-DCP	1	523	0.1	83.8	< LOD	21.5	176.5	1060.3	1935.2	10442.2
	2	479		77.2	< LOD	18.5	158.6	1035.8	1950.0	6063.2
	NHANES	223	0.1	67.3	< LOD	3.7	37.0	187.7	419.3	3077.8

1-Naphthol^a^	1	520	0.2	75.4	0.3	1.9	2.9	5.7	10.8	413.8
	2	479		66.2	< LOD	2.4	3.7	5.8	8.2	167.0
	NHANES	118	6.2 (ng/L)	100^a^	0.9	1.6	3.1	10.8	31.7	297.4

2-Naphthol^a^	1	522	0.4	66.5	< LOD	1.7	5.0	10.9	16.3	45.9
	2	479		68.7	< LOD	1.8	5.4	10.3	15.3	77.9
	NHANES	118	2.4 (ng/L)	100	1.6	3.0	6.1	14.1	22.7	138.7

PNP	1	538	0.1	51.5	< LOD	0.2	0.9	2.2	4.4	44.8
	2	481		58.0	< LOD	0.3	1.0	2.6	4.5	69.1
	NHANES	223	0.1–0.8	30.5	< LOD	< LOD	0.9	2.2	2.9	24.0

ORTH	1	523	0.2	60.6	< LOD	0.5	1.1	1.9	4.3	117.1
	2	479		51.8	< LOD	0.3	1.5	3.2	7.5	67.7
	NHANES	224	0.3	34.4	< LOD	< LOD	0.6	1.7	2.8	19.0

2,4,6-TCP	1	523	0.6	56.2	< LOD	1.4	5.5	11.9	18.2	142.0
	2	478		74.1	0.4	4.5	9.0	17.4	23.4	61.7
	NHANES	223	1–1.3	59.6	< LOD	1.8	4.1	8.6	12.6	68.0

TCPy	1	538	0.3	71.2	< LOD	2.1	5.4	11.4	16.9	56.1
	2	481		81.9	1.0	3.2	7.1	13.4	17.9	38.2
	NHANES	224	0.4	79.0	0.6	1.6	3.1	5.5	7.4	61.0

Abbreviations: DF, detection frequency; Max, maximum. CHAMACOS prenatal sample 1 was collected at approximately 13 weeks gestation, and prenatal sample 2 was collected at approximately 26 weeks gestation; the NHANES prenatal samples were collected at approximately 24 weeks gestation (mean = 24.4 ± 9.5 weeks).

a NHANES detection frequencies for 1- and 2-naphthol become 99% and 93%, respectively, when recalculated using the CHAMACOS LODs.

**Table 4:** Summary of CHAMACOS first and second prenatal and NHANES urinary metabolites (μg/L) with detection frequencies < 50%.

Analyte	Prenatal sample	*n*	LOD (μg/L)	DF (%)	Percentiles			
					75th	90th	95th	Max
AP	1	479	0.3	5.6	< LOD	< LOD	< LOD	46.9
	2	466		6.4	< LOD	< LOD	< LOD	20.7
	NHANES	—	—	—	—	—	—	—

CFP	1	523	0.2	1.2	< LOD	< LOD	< LOD	2.5
	2	479		2.5	< LOD	< LOD	< LOD	14.9
	NHANES	224	0.4	8.9	< LOD	< LOD	0.4	5.1

CIT	1	538	1.5	12.5	< LOD	2.3	6.2	70.9
	2	481		9.2	< LOD	< LOD	2.5	6.8
	NHANES	—	—	—	—	—	—	—

*cis*-DCCA	1	538	0.2	5.6	< LOD	< LOD	0.3	162.8
	2	481		8.7	< LOD	< LOD	0.5	55.7
	NHANES	223	0.1	32.7	0.15	0.4	0.8	40.0

*trans*-DCCA	1	538	0.4	12.5	< LOD	0.5	0.9	397.5
	2	481		16.6	< LOD	0.9	1.5	339.0
	NHANES	223	0.4	26.9	0.4	0.8	1.5	78.0

2,4-D	1	538	0.2	13.8	< LOD	0.3	0.5	8.6
	2	481		21.2	< LOD	0.4	0.8	8.6
	NHANES	221	0.2–1.0	31.2	0.1	0.4	0.5	2.3

DEAMPY	1	538	0.2	5.6	< LOD	< LOD	0.3	29.7
	2	481		4.4	< LOD	< LOD	< LOD	9.1
	NHANES	124	0.2	7.3	< LOD	< LOD	0.6	2.9

DEET	1	538	0.1	5.6	< LOD	< LOD	0.1	1.9
	2	481		1.9	< LOD	< LOD	< LOD	0.3
	NHANES	224	0.1–0.4	17.0	< LOD	0.1	0.2	1.4

DME	1	479	0.03	0.8	< LOD	< LOD	< LOD	1.2
	2	466		3.7	< LOD	< LOD	< LOD	2.0
	NHANES	—	—	—	—	—	—	—

ETU	1	446	0.1	23.5	< LOD	0.7	1.5	14.4
	2	466		7.7	< LOD	< LOD	0.4	11.0
	NHANES	—	—	—	—	—	—	—

4F3PBA	1	538	0.2	3.2	< LOD	< LOD	< LOD	75.2
	2	481		1.9	< LOD	< LOD	< LOD	26.0
	NHANES	222	0.2	1.4	< LOD	< LOD	< LOD	5.6

IMPY	1	538	0.7	2.2	< LOD	< LOD	< LOD	7.7
	2	481		2.7	< LOD	< LOD	< LOD	7.3
	NHANES	222	0.7–7.0	11.7	< LOD	1.3	1.8	12.0

MDA	1	359	0.3	32.0	0.5	1.3	2.3	57.5
	2	265		26.4	0.4	1.3	1.9	45.1
	NHANES	92	≤ 2.6	41.3	0.6	0.9	1.8	7.4

MET	1	538	0.2	7.3	< LOD	< LOD	0.23	0.3
	2	481		1.9	< LOD	< LOD	< LOD	0.5
	NHANES	127	0.2	1.6	< LOD	< LOD	< LOD	0.3

PCP	1	523	0.9	4.0	< LOD	< LOD	< LOD	8.3
	2	479		2.3	< LOD	< LOD	< LOD	29.5
	NHANES	224	0.3–0.5	10.3	< LOD	0.4	1.5	15.2

3PBA2	1	538	0.1	21.4	< LOD	0.5	0.9	222.6
	2	481		27.0	0.1	0.5	1.1	225.0
	NHANES	224	0.1	67.0	0.5	1.3	2.2	58.7

2,4,5-TCP	1	517	1.0	45.1	7.9	45.6	203.1	2145.5
	2	479		48.9	8.3	37.4	65.2	554.1
	NHANES	224	0.9	24.1	0.6	2.5	5.0	95.5

Abbreviations: DF, detection frequency; Max, maximum. CHAMACOS prenatal sample 1 was collected at approximately 13 weeks gestation, and prenatal sample 2 was collected at approximately 26 weeks gestation. NHANES samples were collected around 24 weeks gestation (mean = 24.4 ± 9.5 weeks). ACET, ATZ, CMHC, DBCA, IPP, MMP, OMT, PTU, and 2,4,5-T, were not included because these compounds were detected in < 3% of prenatal samples (DF ≤ 3%).
